# Transcatheter Arterial Chemoembolization Combined with Hepatic Arterial Infusion Chemotherapy Versus Transcatheter Arterial Chemoembolization for Unresectable Hepatocellular Carcinoma: A Systematic Review and Meta-analysis

**DOI:** 10.5152/tjg.2024.23228

**Published:** 2024-04-01

**Authors:** Guoying Feng, Yi Feng, Shu Yao, Xun Huang, Zuxiang Peng, Yongliang Tang, Wen Tang, Zhengyan Li, Hanchen Wang, Hongming Liu

**Affiliations:** Department of Hepatobiliary Surgery, Daping Hospital, Army Medical University, Chongqing, China

**Keywords:** Transcatheter arterial chemoembolization, hepatic arterial infusion chemotherapy, hepatocellular carcinoma, combination therapy, meta-analysis

## Abstract

Background/Aims: In this study, we evaluated the efficacy and safety of transcatheter arterial chemoembolization (TACE) combined with hepatic arterial infusion chemotherapy (HAIC) compared to TACE monotherapy for the treatment of unresectable hepatocellular carcinoma (HCC).

Materials and Methods: Relevant studies were systematically searched in PubMed, Embase, Web of Science, and Cochrane Library databases until September 1, 2023. Our analysis included 7 cohort studies encompassing a total of 630 patients.

Results: The results demonstrated that the TACE plus HAIC group exhibited significantly improved prognosis compared to the TACE alone group, as evidenced by superior rates of complete response, partial response, progressive disease, objective response rate, and disease control rate. Moreover, the TACE group displayed a lower risk of platelet reduction and vomiting when compared to the TACE plus HAIC group. None of the 7 studies reported any intervention-related mortality.

Conclusion: In conclusion, the combination of TACE and HAIC may be recommended as a viable option for patients with unresectable HCC, given its evident enhancements in survival and tumor response rates without significant differences in adverse events when compared to TACE monotherapy. Nevertheless, additional randomized controlled trials and studies involving Western cohorts are warranted to further validate these findings.

**Main Points**

- In the management of unresectable hepatocellular carcinoma, the combined application of transcatheter arterial chemoembolization (TACE) and hepatic arterial infusion chemotherapy (HAIC) yielded significantly improved survival outcomes and tumor response rates compared to TACE monotherapy.
- The overall incidence of adverse events was comparable between patients receiving TACE treatment and those receiving combined TACE + HAIC therapy, while the TACE group exhibited a lower occurrence of vomiting.
- The types of literature included might have influenced the conclusions drawn, thus emphasizing the need for further validation through future randomized controlled trials.

## Introduction

Hepatocellular carcinoma (HCC) represents a highly prevalent malignant tumor and stands as the third leading cause of cancer-related mortality worldwide.^1,2^ For patients who have the opportunity to undergo radical treatment, hepatectomy, and liver transplantation are considered optimal choices, with ongoing refinements aimed at minimizing morbidity and mortality.^3^ Nevertheless, due to its insidious onset and rapid progression, most patients are diagnosed with advanced-stage disease, making them ineligible for curative surgical interventions.^4^ As a result, alternative strategies are indispensable for individuals with unresectable HCC.

Systematic therapies, such as molecular-targeted agents (MTAs) and immune checkpoint inhibitors (ICIs), alongside local treatment modalities including transcatheter arterial chemoembolization (TACE), hepatic arterial infusion chemotherapy (HAIC), and radiation therapy have been demonstrated to significantly enhance therapeutic outcomes for patients with unresectable HCC.^5-8^ Notably, 8%-18% of patients have shown promising results, with the potential for hepatectomy^9^ or extended median survival of up to 47.7 months following TACE treatment.^10^ This is attributed to the effective occlusion of tumor blood supply vessels, which increases drug concentration and prolongs drug efficacy.^11,12^ In parallel, HAIC ensures sustained high local concentrations of chemotherapeutic agents within tumors without embolization. Recognizing the limited clinical efficacy of individual strategies alone, various combinational or sequential treatment approaches have been explored and validated, demonstrating beneficial prognostic impact for unresectable HCC.^13-15^ Prominently featured among intra-arterial therapies, TACE and HAIC are widely utilized in distinct combination strategies.^14,15^ Recent studies, particularly in East Asia, have investigated the efficacy and safety of combining TACE and HAIC for unresectable HCC.^16-18^ Comparative to TACE monotherapy, the synergistic effects of TACE plus HAIC not only induce partial tumor necrosis and diminish tumor volume by impeding blood supply but also enhance antitumor immune responses triggered by the resulting necrotic tissue. Overall, the independent antitumor mechanisms of chemoembolization, combined with the localized high drug concentration achieved through HAIC, may collectively achieve superior therapeutic effects and reduce the risk of chemotherapy resistance-associated tumor progression.^19^

Although previous studies have provided supporting evidence for the efficacy and safety of the TACE plus HAIC strategy,^16-18^ a comprehensive understanding of the advantages and disadvantages of combination treatment compared to TACE monotherapy remains elusive without compelling randomized controlled trials (RCTs). Additionally, concerns exist regarding the potential reduction in the diffusion of chemotherapeutic drugs during subsequent HAIC, which could theoretically impact treatment outcomes. Due to the limited number of studies available for direct comparison of the efficacy and safety between TACE plus HAIC and TACE alone, this meta-analysis aims to provide a more robust evaluation of the therapeutic benefits attained with TACE plus HAIC versus TACE monotherapy in the management of unresectable HCC by including all relevant studies currently available.

## Materials and Methods

This present study, comprising a systematic review and meta-analysis, adhered meticulously to the Preferred Reporting Items for Systematic Reviews and Meta-Analyses (aka PRISMA) guidelines as recommended by the Cochrane Handbook.^20^ Furthermore, the study protocol was registered in the PROSPERO international database (Registration No. CRD42023411685, available at https://www.crd.york.ac.uk/prospero/).

### Literature Retrieval Process

We conducted a comprehensive search using the PubMed, Embase, Web of Science, and Cochrane Library databases to identify relevant studies for inclusion in this analysis. The retrieval cutoff date was September 1, 2023. The retrieval strategy was based on Medical Subject Headings (i.e., MeSH) terms of “liver cancer” AND (“TACE” OR “transcatheter arterial chemoembolization” OR “chemoembolization” OR “embolization”) AND (“hepatic arterial infusion chemotherapy” OR “infusion”) and free terms of them. In addition to the primary database searches, we conducted a thorough examination of the reference lists of all included studies to identify any additional relevant data that may have been missed by the initial search.

### Study Selection and Eligibility Criteria

In our study, the term “unresectable HCC” encompassed large tumors with inadequate future liver remnant or advanced tumors demonstrating main portal invasion and/or portal vein tumor thrombus (PVTT). We established specific inclusion criteria as follows: (i) RCTs or observational studies; (ii) inclusion of patients diagnosed with unresectable HCC; (iii) comparison of patients’ clinical outcomes between receiving TACE plus HAIC and TACE alone, without any other concurrent interventions; (iv) reporting of clinical outcomes such as overall survival (OS), progression-free survival (PFS), response rates, and adverse events for comparative analysis; (v) inclusion of human studies published exclusively in English.

Exclusion criteria were applied as follows: (i) absence of a control group; (ii) Conference publications, letters, reviews, case reports, comments, unpublished or incomplete studies, or duplicate data; (iii) non-English publications or redundant studies; (iv) Liver metastases originating from any other primary tumor site. Any discrepancies pertaining to the selection of eligible studies were resolved through discussions among the investigators.

### Data Extraction

Data extraction was performed independently by 2 reviewers using standardized forms. The following information was extracted from each study: the last name of the first author, publication date, study design, number of patients, patients’ demographic characteristics including gender, age, hepatitis B virus (HBV) history, Child–Pugh classification, Barcelona Clinic Liver Cancer (BCLC) staging, tumor size and number, details of TACE and HAIC treatment regimens employed in each study (including drug and dosage), hazard ratios (HRs) for OS and PFS, assessment of tumor response according to Modified Response Evaluation Criteria in Solid Tumors, encompassing complete response (CR), partial response (PR), stable disease (SD), progressive disease (PD), objective response rate (ORR), disease control rate (DCR), documentation of adverse events, and intervention-related mortality. If HR data were unavailable from the article or authors, Engauge Digitizer V4.1 software, along with previously described method,^21^ was utilized to calculate HR values from Kaplan–Meier survival curves. In cases where discrepancies or disagreements arose during the data extraction process, a third reviewer was consulted to facilitate resolution through discussion.

### Quality Assessment

The Newcastle–Ottawa Scale,^22^ specifically chosen to align with the types of studies included in our analysis, was employed for conducting quality assessments. Independent evaluations of study quality were performed by 2 reviewers, assigning a scoring system ranging from 0 to 9 stars for each study. Scores of 0-3, 4-6, and 7-9 were interpreted as indicative of low, moderate, and high quality, respectively. In the event of any discrepancies, a third reviewer’s expertise was sought to provide guidance and facilitate resolution.

### Statistical Analysis

The overall pooled HR and corresponding 95% CI for patients’ OS and PFS were calculated using the inverse variance method, as previously described.^21^ Comparative studies were assessed using risk ratios (RRs) with a 95% CI. A random effects model was employed for all data analyses. Statistical significance was defined as *P* < .05 with the 95% CI for the pooled HR and RR not overlapping 1. Heterogeneity was evaluated using *I*^2^ statistics and the *Q* test. Subgroup or sensitivity analyses were conducted when appropriate to identify potential sources of heterogeneity. If the number of included studies exceeded 10, publication bias was assessed through visual inspection of funnel plots and by performing the rank correlation test of Begg and the regression asymmetry test of Egger. Review Manager 5.4.1 software was utilized for all statistical analyses.

## Results

### Study Characteristics and Quality

**Figure 1.**
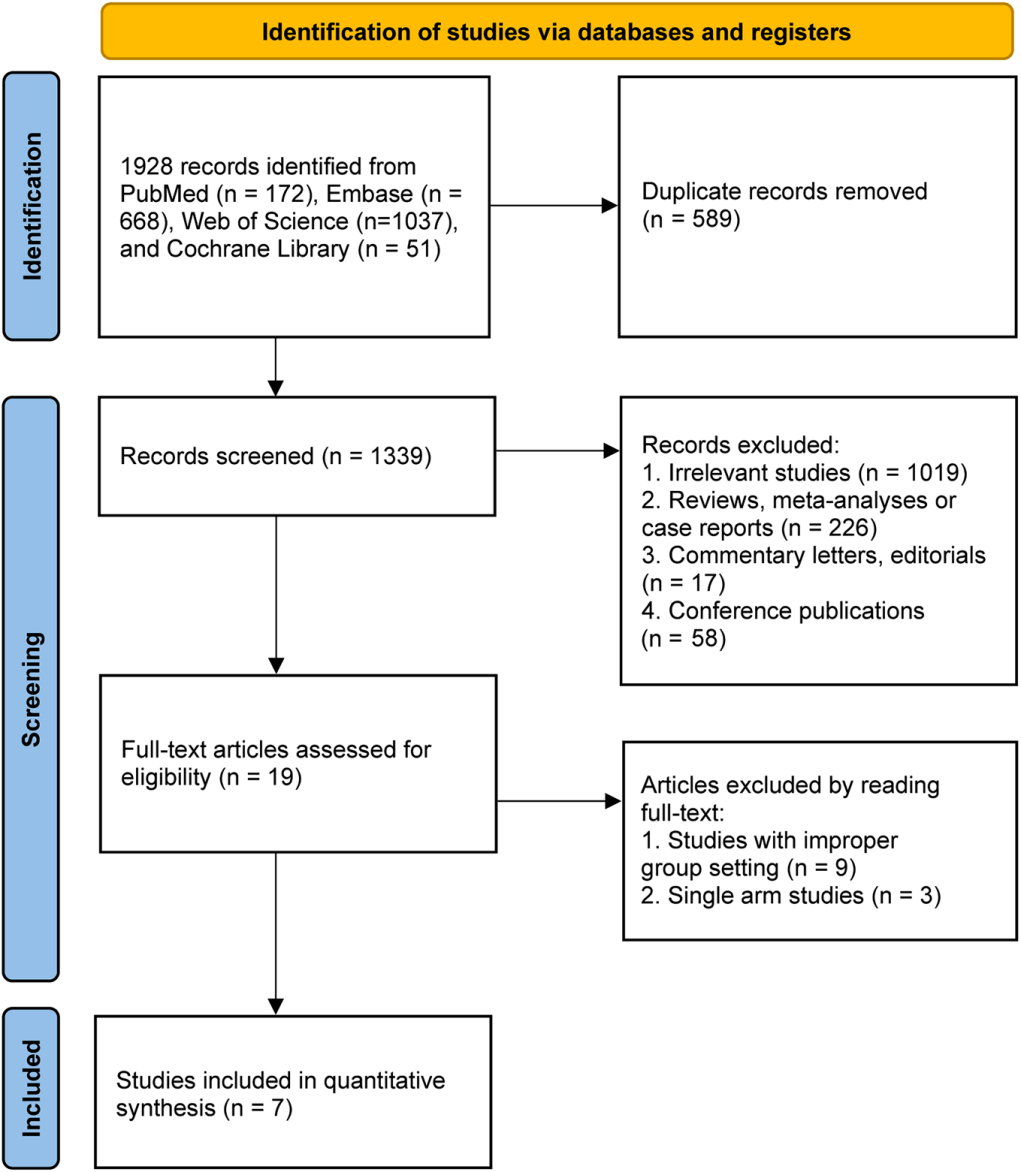
The flowchart of study selection.

A total of 1928 studies were initially retrieved, from which 589 duplicates were identified and removed. Subsequently, 1320 studies were excluded due to irrelevance or inappropriate article format, leaving 19 full-text articles for further assessment of eligibility. After further screening, 12 articles were excluded based on inadequate study design. Consequently, a final selection of 7 cohort studies was included in the meta-analysis,^23-29^ as depicted in Figure 1. All included studies were cohort studies, comprising 1 prospective study and 6 retrospective studies conducted in East Asia (1 from South Korea and 6 from China), published between 2013 and 2022. The meta-analysis encompassed data from 7 studies involving 630 patients (313 in the TACE plus HAIC group and 317 in the TACE group). Comprehensive information regarding the baseline characteristics and intervention details of the included studies can be found in Tables 1 and 2.^23-29^ Quality assessment results of the included studies are presented in Supplementary Table 1, demonstrating the absence of any notably inferior-quality studies. The comprehensive details regarding the inclusion criteria for patients included in each study can be found in Supplementary Table 2.

### Primary Outcomes

**Figure 2.**
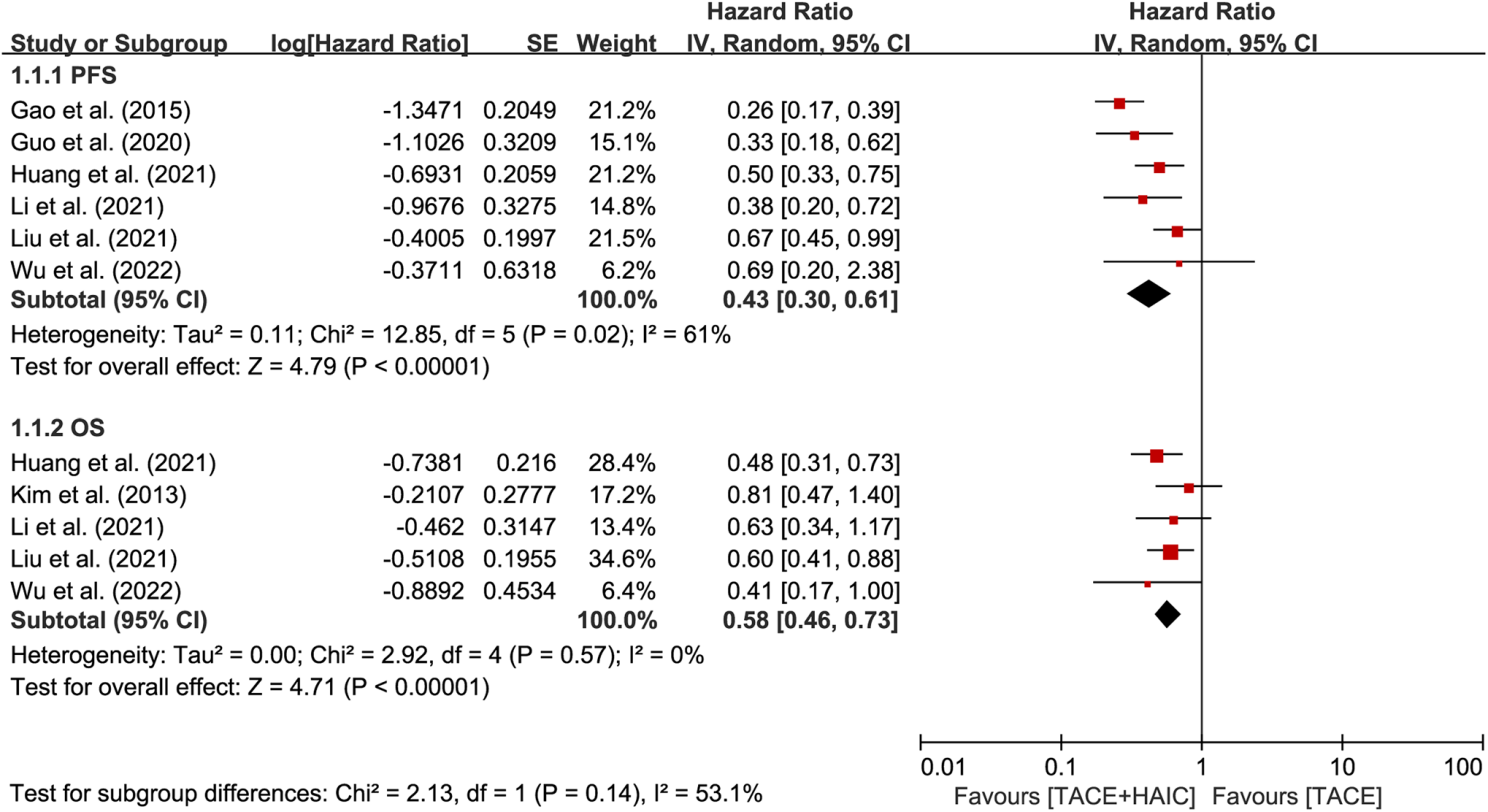
Forest plots of survival between transcatheter arterial chemoembolization (TACE) plus hepatic arterial infusion chemotherapy group and TACE group.

The primary outcomes of interest encompassed essential clinical efficacy indicators, including PFS, OS, and tumor response. Progression-free survival data were reported in 6 studies,^24-29^ while OS data were available in 5 studies.^23,26-29^ The HRs for PFS and OS are presented in Figure 2. The findings unequivocally demonstrated that patients in the TACE plus HAIC group exhibited significantly improved prognosis compared to those in the TACE group (PFS: HR = 0.43, 95% CI 0.30-0.61, *P* < .00001,* I*^2^ = 61%; OS: HR = 0.58, 95% CI 0.46-0.73, *P* < .00001, *I*^2^ = 0).

**Figure 3.**
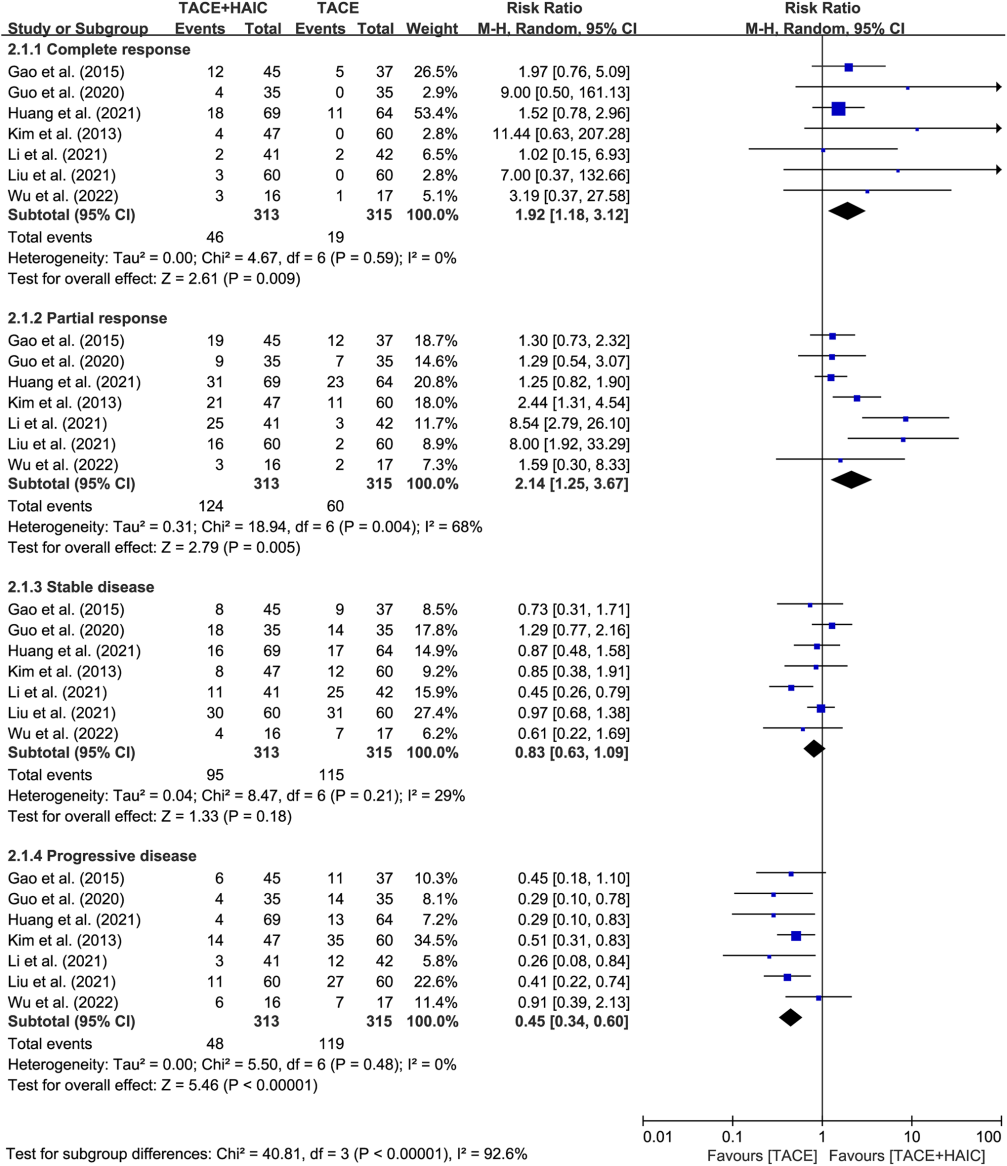
Forest plots of complete response, partial response, stable disease, and progressive disease between transcatheter arterial chemoembolization (TACE) plus hepatic arterial infusion chemotherapy group and TACE group.

**Figure 4.**
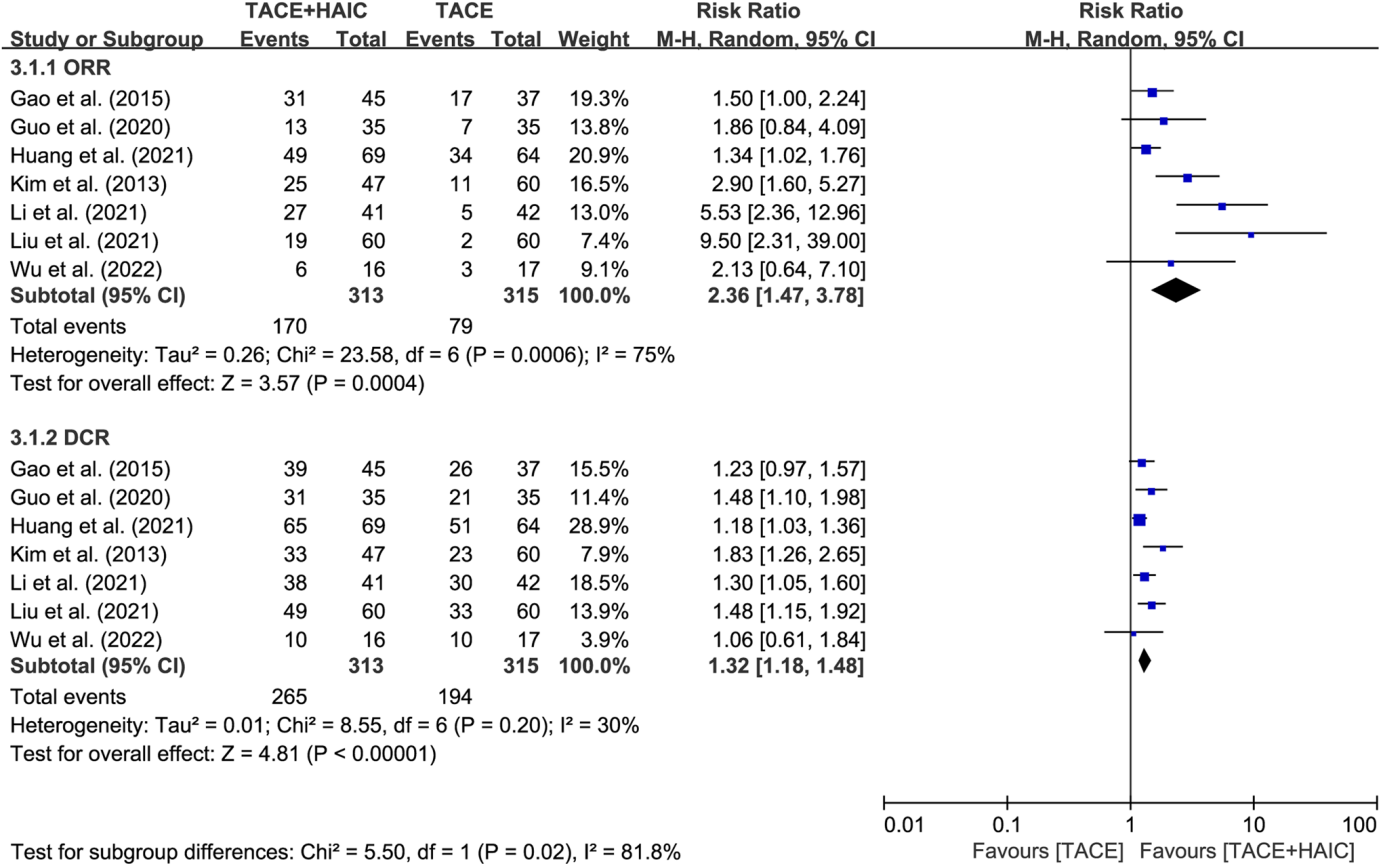
Forest plots of objective response rate (ORR) and disease control rate (DCR) between transcatheter arterial chemoembolization (TACE) plus hepatic arterial infusion chemotherapy (HAIC) group and TACE group.

All 7 included studies provided tumor response data following treatment. The pooled analysis demonstrated clear advantages for patients in the TACE plus HAIC group regarding CR, PR, and PD rates (CR: RR = 1.92, 95% CI 1.18-3.12, *P* = .009, *I*^2^ = 0; PR: RR = 2.14, 95% CI 1.25-3.67, *P* = 0.005, *I*^2^ = 68%; PD: RR = 0.45, 95% CI 0.34-0.60, *P* < 0.00001, *I*^2^ = 0) (Figure 3). However, no significant difference was observed between the 2 groups in terms of SD rate (RR = 0.83, 95% CI 0.63-1.09, *P* = 0.18, *I*^2^ = 29%) (Figure 3). Similar advantages were found in both groups for ORR and DCR (ORR: RR = 2.36, 95% CI 1.47-3.78, *P* = 0.0004, *I*^2^ = 75%; DCR: RR = 1.32, 95% CI 1.18-1.48, *P* < 0.00001, *I*^2^ = 30%) (Figure 4).

### Secondary Outcomes

Secondary outcomes encompassed adverse events and intervention-related mortality. Regarding routine blood examination, 5 studies^24-27,29^ reported myelosuppression, including reductions in red blood cells, white blood cells, and platelets. Notably, thrombocytopenia emerged as the sole differentiating indicator between the 2 groups, with patients in the TACE group exhibiting a lower risk of platelet reduction compared to those in the TACE plus HAIC group (RR = 1.59, 95% CI 1.25-2.02, *P* = .0002, *I*^2^ = 0). Furthermore, 5 studies^24-27,29^ reported elevations in alanine aminotransferase (ALT), while 4 studies^24,25,27,29^ reported elevations in aspartate aminotransferase (AST). However, no significant differences were observed between the 2 groups (ALT: RR = 1.09, 95% CI 0.85-1.40, *P* = .49, *I*^2^ = 73%; AST: RR = 0.98, 95% CI 0.85-1.12, *P* = .75, *I*^2^ = 19%). Similarly, a comparison of bilirubin elevation among 6 studies^24-29^ revealed no significant difference between the 2 groups (RR = 1.13, 95% CI 0.92-1.40, *P* = .25, *I*^2^ = 65%).

**Figure 5.**
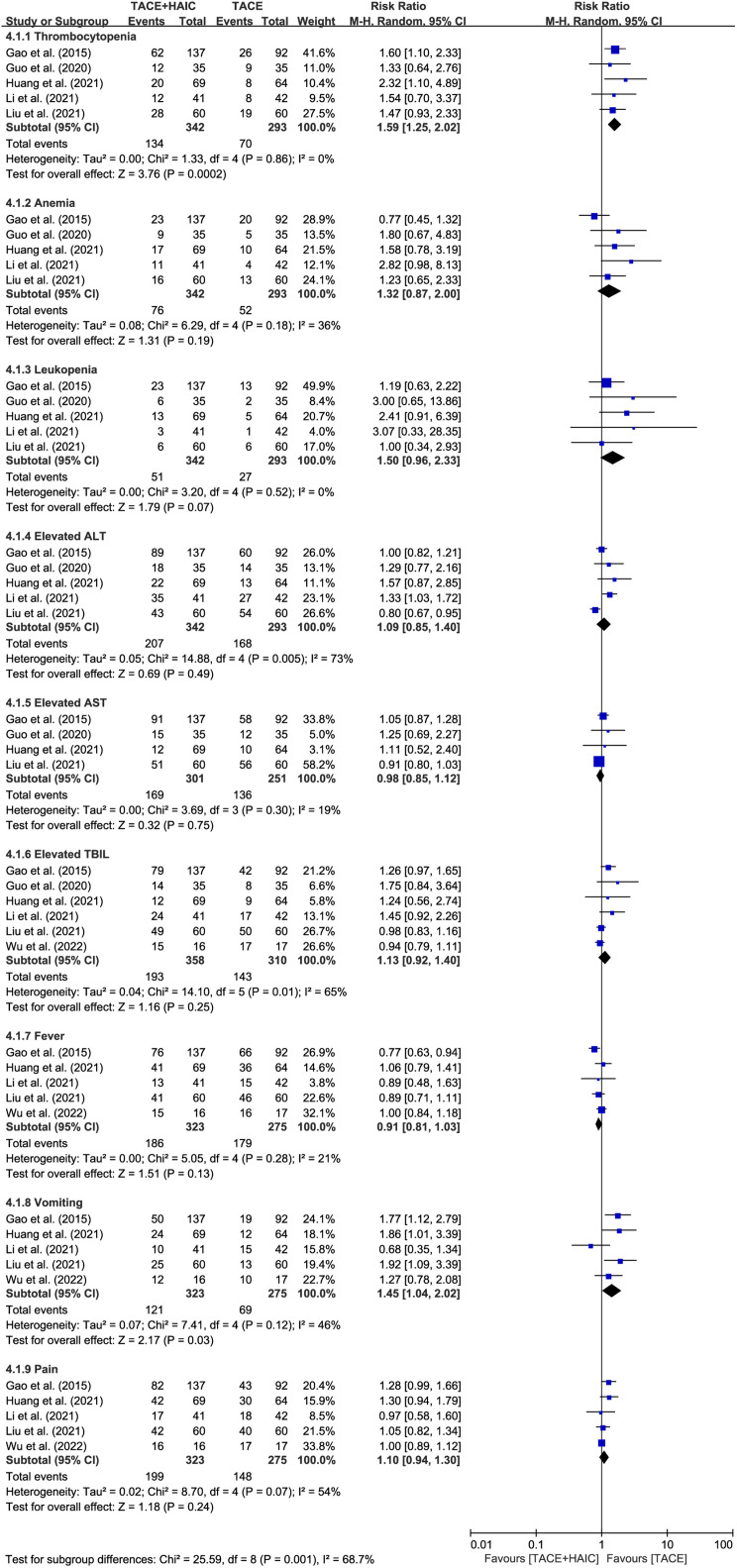
Forest plots of adverse events between transcatheter arterial chemoembolization (TACE) plus hepatic arterial infusion chemotherapy (HAIC) group and TACE group. ALT, alanine aminotransferase; AST, aspartate aminotransferase; TBIL, total bilirubin.

The incidence of adverse events, including fever, vomiting, and pain, was reported in 5 studies.^24,26-29^ Interestingly, the incidence of vomiting was significantly lower in the TACE group than in the TACE plus HAIC group (RR = 1.45, 95% CI 1.04-2.02, *P* = .03, *I*^2^ = 46%, Figure 5); however, no significant differences were observed in the incidence of fever and pain between the 2 groups (fever: RR = 0.91, 95% CI 0.81-1.03, *P* = .13, *I*^2^ = 21%; pain: RR = 1.10, 95% CI 0.94-1.30, *P* = .24, *I*^2^ = 54%, Figure 5). Notably, intervention-related mortality was not reported in any of the 7 included studies.

### Sensitivity and Subgroup Analysis

A sensitivity analysis was performed by systematically excluding each study from the analysis, one at a time, to assess the influence of individual studies on the overall results. The pooled HRs and corresponding 95% CIs for PFS, OS, and each indicator of tumor response exhibited no significant variation upon exclusion of any specific study.

Considering variations in dosing regimens of HAIC and the inclusion or exclusion of PVTT among the included studies, subgroup analyses were conducted to compare HCC with PVTT subgroup, various unresectable HCC subgroup, and cisplatin/FOLFOX subgroup. The results of the cisplatin and FOLFOX subgroups were consistent with the sensitivity analysis upon excluding Kim et al’s^23^ study, as Kim et al’s^23^ study was the sole study employing the cisplatin regimen. In both the HCC with PVTT subgroup and various unresectable HCC subgroup, the tendencies observed in OS, PFS, SD, and DCR remained consistent with the findings in Primary Outcomes. However, there were no significant differences observed in CR, PR, PD, and ORR between patients treated with TACE plus HAIC and those receiving TACE alone in the HCC with PVTT subgroup (CR: RR = 4.20, 95% CI 0.74-23.91, *P* = .11, *I*^2^ = 0; PR: RR = 3.77, 95% CI 0.75-19.04, *P* = .11, *I*^2^ = 55%; PD: RR = 0.58, 95% CI 0.26-1.26, *P* = .17, *I*^2^ = 57%; ORR: RR = 4.31, 95% CI 0.92-20.22, *P* = .06, *I*^2^ = 64%) (Table 3).

## Discussion

Transcatheter arterial chemoembolization has emerged as a notable therapeutic strategy for advanced HCC, demonstrating considerable advancements in its efficacy.^9,30^ Additionally, studies have reported significant benefits of HAIC with the FOLFOX regimen (oxaliplatin, fluorouracil, and leucovorin) for patients with advanced HCC, particularly those with large tumors or PVTT.^31-33^ Several investigations have sought improved clinical outcomes by investigating the combination of TACE or HAIC with other therapies such as MTAs and ICIs, some of which have exhibited practical feasibility.^34,35^ Notably, HAIC has been shown to significantly improve OS compared to TACE alone in patients with unresectable HCC.^36^ However, limited attention has been given to the combination of these 2 therapeutic strategies.

This meta-analysis encompassed 7 cohort studies involving a total of 630 patients, aiming to evaluate the efficacy and safety of combining TACE and HAIC versus TACE alone in unresectable HCC. The findings revealed that patients treated with TACE plus HAIC demonstrated improved survival outcomes, enhanced tumor response rates, and comparable risks of adverse events compared to those receiving TACE alone. Notably, previous single-arm studies have also reported on the efficacy of TACE plus HAIC,^16-18^ suggesting that this combined treatment modality may emerge as another viable option for individuals with unresectable HCC. A combination strategy such as this offers numerous advantages: TACE disrupts tumor blood flow, leading to prolonged exposure of the tumor to high concentrations of chemotherapy drugs. Consequently, hypoxic and ischemic conditions induced within the tumor tissue impair transmembrane ion pump function, reducing drug efflux from the tumor.^27,37^ Subsequent HAIC can further augment and sustain elevated drug concentrations, facilitating the elimination of residual lesions. Moreover, employing different drug regimens for TACE and HAIC may enhance treatment efficacy against drug-resistant tumors. Recent evidence suggests that the combination of TACE and HAIC may be particularly beneficial in cases involving difficult-to-locate and embolize fine-spun tumor-feeding arteries.^25^ Furthermore, vessel co-option, which involves the utilization of normal arteries by tumor cells, appears to play a critical role in tumor development, progression, and resistance to various MTAs.^38-40^ The conversion rate to resection represents another important indicator for evaluating clinical efficacy. However, only 2 studies definitively reported data on conversion rates. One study demonstrated significant superiority of the TACE plus HAIC group over the TACE group [48.8% (20/41) vs. 9.5% (4/42), *P* < .001].^26^ In another single-arm study, 8.3% (11/132) of patients achieved resection following TACE plus HAIC treatment.^16^ Unfortunately, no other included studies provided conversion rate data for TACE plus HAIC treatment.

Previous meta-analyses have indicated that TACE may be associated with a higher incidence of adverse events, such as fever and elevations in serum ALT and bilirubin levels, compared to HAIC.^41^ It was anticipated that TACE plus HAIC treatment might carry a significantly increased risk of adverse events when compared to TACE monotherapy. However, our meta-analysis did not reveal a substantial elevation in the risk of adverse events in the TACE plus HAIC group compared to the TACE-only group. Among the observed differences, thrombocytopenia and vomiting were the only discernible effects that could be attributed to the administration of additional chemotherapeutic drugs. In light of the potential superiority of TACE plus HAIC over TACE, the occurrence of these relatively mild adverse events associated with the combination regimen can be completely acceptable for clinicians and effectively managed accordingly. Additionally, it is plausible that patients with relatively preserved hepatic function, as reflected by the inclusion of 254 Child–Pugh stage A and 20 stage B patients out of the 274 data-accessible patients in our analysis, may exhibit improved tolerance to adverse events. Thus, the meticulous selection of appropriate patients likely contributed significantly to this outcome. Considering the potential occurrence of postembolization syndrome, characterized by symptoms such as fever, abdominal pain, and nausea/vomiting^42^ as well as the myelosuppression associated with chemotherapeutic agents, the implementation of rigorous patient selection criteria prior to initiating this combination treatment may be warranted.

In this study, numerous merged results exhibit a substantial degree of heterogeneity. This can be ascribed to several factors. First, the limited number of included studies and cases within our analysis contributes to this heterogeneity. Furthermore, variations among the included studies, particularly in terms of diverse treatment regimens and intervals for TACE, further contribute to the observed heterogeneity. Despite TACE being an established therapeutic modality for liver cancer, the presence of drug resistance and the inherent heterogeneity within HCC pose challenges in determining the optimal treatment regimen. Consequently, personalized treatment strategies are frequently adopted in clinical practice, leading to notable discrepancies in drug combinations among different medical centers. Numerous studies^24,25,43^ underscore the significance of embolization as a fundamental and integral component of combination therapy for high-burden tumors. We posit that this importance is particularly heightened in the context of the TACE plus HAIC regimen. Embolization plays a pivotal role in inducing ischemic and hypoxic effects within the tumor tissue as well as impairing transmembrane ion pumps in tumor cells during the TACE stage. Subsequent administration of HAIC ensures sustained and elevated concentrations of chemotherapeutic agents, thereby minimizing the impact of chemotherapy drugs administered during the TACE phase. The minimal heterogeneity observed in OS, CR, and other outcome measures further strengthens the robustness and validity of our conclusions.

The potential source of heterogeneity in this meta-analysis also lies in the distinction of HCC patients with or without PVTT. Given the lack of essential data, our ability to subgroup the included studies was limited to distinguishing between those exclusively involving HCC patients with PVTT and all unresectable HCC patient populations. Contrary to the findings of the overall analysis, the subgroup analysis revealed no significant differences in terms of CR, PR, PD, or ORR between the TACE plus HAIC and TACE-only groups in HCC with PVTT subgroup. However, heterogeneity persisted within this subgroup. Although our findings are impacted by the limited number of studies and relatively ambiguous subgrouping, the suitability of TACE plus HAIC treatment for HCC patients with PVTT warrants careful consideration. According to guidelines from the Japan Society of Hepatology and recent studies, HCC with PVTT should not be regarded as a contraindication for TACE and may even benefit from it in terms of survival.^27,44^ Notably, emerging evidence underscores the substantial ORR and OS achieved through combined treatment with TACE plus HAIC, particularly when compared to various forms of systemic therapy such as MTAs and ICIs.^18^ Therefore, we maintain an optimistic stance while eagerly anticipating further studies in the field.

The exploration of combined local therapies for HCC extends beyond the TACE + HAIC regimen, with increasing research focusing on the safety and efficacy of various combination approaches. Given that TACE inevitably leads to tumor hypoxia-induced damage and enhanced expression of circulating or tissue vascular endothelial growth factor (VEGF), the addition of anti-angiogenic agents may exert complementary inhibitory effects on neovascularization and tumor growth.^45^ Liu et al^46^ combined sorafenib with TACE–HAIC, resulting in 6-month, 12-month, and 24-month PFS rates of 75.0%, 54.7%, and 30.0%, respectively, demonstrating significant efficacy and good tolerability in unresectable HCC patients. Yuan et al^47^ combined TKIs with PD-1 inhibitors in patients with HCC and PVTT who underwent TACE–HAIC, and the results showed improved survival outcomes, pathological CR rates, and conversion rates compared to TACE monotherapy. Drawing on the same rationale, a meta-analysis^48^ has assessed the effectiveness of the combined therapeutic approach involving HAIC in conjunction with sorafenib. The outcomes unveiled that this therapeutic regimen not only heightens ORRs but also prolongs OS, while maintaining an acceptable tolerability profile in relation to adverse events. Similarly, Chen et al^43^ employed a combination therapy approach involving HAIC, lenvatinib, tislelizumab, and transarterial embolization for HCC patients with PVTT, demonstrating its safety and efficacy in patients with high tumor burden.

Transarterial radioembolization (TARE) is a procedure involving the intra-arterial injection of yttrium-impregnated glass microspheres or resin microspheres. Some studies^49,50^ have reported similar or even better results in mid-term HCC treatment with TARE compared to TACE, particularly in terms of time to progression. However, reports on combined treatments involving TARE are currently limited, and a prospective phase III trial^51^ investigating its superiority in combination with sorafenib failed to establish its advantage, underscoring the need for further exploration. In terms of systemic therapy, the combination of atezolizumab (anti-PD-L1 antibody) and bevacizumab (anti-VEGF antibody) is the preferred first-line treatment.^5^ However, there have been few reports on their combination with intra-arterial therapies, potentially due to the selective nature of this approach for patients. To benefit from the Atezo–Bev regimen, patients must have preserved liver function (compensated Child–Pugh A if underlying cirrhosis is present) and the absence of high-risk bleeding stigmata on upper endoscopy.^52^ On the other hand, the TACE–HAIC regimen offers a relatively broader range of patient selection, which can be considered an advantage.

This represents the first comprehensive study to date that systematically evaluates the efficacy and safety of TACE plus HAIC compared to TACE monotherapy. The results exhibit a remarkable degree of stability, as confirmed by sensitivity analyses and subgroup evaluations. However, it is important to acknowledge certain limitations in the current body of evidence: i) the relatively small number of included studies (n = 7); ii) the exclusive focus on studies conducted in East Asia; and iii) potential variations in the definition of unresectable HCC among the included studies, albeit minor. Moreover, recent research has highlighted the significant improvement in OS achieved with HAIC in comparison to TACE for patients with unresectable HCC.^36^ Consequently, a comparison between the efficacy of TACE plus HAIC and HAIC alone holds considerable interest; however, to date, no eligible studies addressing this comparison have been identified. Therefore, the necessity for further clinical trials becomes evident, warranting future investigations in this domain.

In conclusion, our findings demonstrate that the utilization of TACE plus HAIC as a treatment modality for patients with unresectable HCC yields superior survival outcomes and tumor response rates compared to TACE monotherapy, while maintaining a comparable incidence of adverse events. This combined therapeutic strategy holds promise in further enhancing the OS and conversion rate for individuals with unresectable HCC. However, it is important to note that the current body of evidence primarily consists of studies conducted in East Asia, highlighting the need for additional RCTs and data from Western cohorts to validate these results in more diverse populations.

## Figures and Tables

**Table 1.** Basic Characteristics of Included Studies in the Meta-analysis

Study	Country	Study Design	Group	Patients	Gender (Male/Female)	Age	HBV (Yes/No)	Child–Pugh (A/B)	BCLC (A/B/C)	Tumor Size (cm)		Number of Tumors	
										≤10	>10	>3	≤3
Kim et al^23^	KOR	Retrospective	TACE	60	54/6	55.30 ± 9.40	50/10	N/A	N/A	24	36	N/A	N/A
			TACE + HAIC	47	42/5	53.70 ± 9.60	40/7	N/A	N/A	19	28	N/A	N/A
Gao et al^24^	CHN	Prospective	TACE	39	35/4	59.69 ± 13.13	33/3	36/3	4/9/22	19	20	22	17
			TACE + HAIC	45	41/4	57.16 ± 10.34	42/0	41/4	3/15/21	29	16	31	14
Guo et al^25^	CHN	Retrospective	TACE	35	33/2	51.60 ± 12.90	31/4	31/4	1/10/24	N/A	N/A	N/A	N/A
			TACE + HAIC	35	32/3	47.70 ± 10.90	33/2	34/1	1/10/24	N/A	N/A	N/A	N/A
Liu et al^26^	CHN	Retrospective	TACE	60	51/9	53.63 ± 11.47	54/3	56/4	N/A	35	25	N/A	N/A
			TACE + HAIC	60	53/7	54.67 ± 9.47	53/4	56/4	N/A	37	23	N/A	N/A
Wu et al^28^	CHN	Retrospective	TACE	17	17/0	48.59 ± 9.99	16/1	N/A	N/A	11.67 ± 5.17		15	2
			TACE + HAIC	16	16/0	47.63 ± 11.06	14/2	N/A	N/A	12.09 ± 5.35		13	3
Li et al^27^	CHN	Retrospective	TACE	42	37/5	≥60, n = 17	33/9	N/A	N/A	21	21	13	29
			TACE + HAIC	41	30/11	≥60, n = 11	36/5	N/A	N/A	18	23	13	28
Huang et al^29^	CHN	Retrospective	TACE	64	55/9	51 (42-62) *	58/6	N/A	7/25/32	36	28	23	41
			TACE + HAIC	69	62/7	55 (44-62) *	65/4	N/A	4/26/39	34	35	30	39

BCLC, Barcelona Clinic Liver Cancer; HAIC, hepatic arterial infusion chemotherapy; HBV, hepatitis B virus; N/A, not available; TACE, transcatheter arterial chemoembolization.

*Median (interquartile range).

**Table 2.** Intervention Details of Included Studies

Study	Group	Drugs and Dosage	Interval	Termination
Kim et al^23^	TACE	Iodized oil (2-12 mL), doxorubicin hydrochloride (10-60 mg), and gelatin sponge particles mixed with 2 mg of mitomycin C.	N/A	N/A
	TACE + HAIC	After TACE. Cisplatin (50-100 mg) was diluted to 0.5 mg/mL and infused at a rate of 4-10 mL/min after TACE.	N/A	N/A
Gao et al^24^	TACE	EPI 40 mg, lipiodol (< 20 mL), and PVA for chemoembolization treatment.	N/A	N/A
	TACE + HAIC	After TACE. OXA: 60-75 mg/m^2^ pumped after 0-4 hours via artery; CF: 200 mg/m^2^, pumped after 2-4 hours intravenous; 5-FU:1-1.5 g/m^2^, pumped after 4-24 hours via artery.	N/A	N/A
Guo et al^25^	TACE	OXA (100 mg/m^2^), leucovorin (200 mg/m^2^), 5-FU (400 mg/m^2^), and gelatin sponge or embospheres.	N/A	Until progressive disease was indicated by multiphase CT during the follow-up of 24 months in both groups.
	TACE + HAIC	After TACE. Day 1: OXA (100 mg/m^2^, 2 hours), leucovorin (200 mg/m^2^, 2 hours), 5-FU (400 mg/m^2^, 15 minutes), 5-FU (600 mg/m^2^, 22 hours). Day 2: leucovorin (200 mg/m^2^, 2 hours), 5-FU (400 mg/m^2^, 15 minutes), 5-FU (600 mg/m^2^, 22 hours).	3 weeks	
Liu et al^26^	TACE	Lipiodol (5-15 mL), EPI (40-60 mg), and gelatin sponge particles.	N/A	Until the intrahepatic lesions progressed or toxicity became unacceptable.
	TACE + HAIC	After TACE. OXA (85 mg/m^2^) administered intra-arterially for 4 hours, leucovorin (200 mg/m^2^) administered intravenously for 2 hours, and 5-FU (1.5 g/m^2^) administered intra-arterially for 20 hours.	6-8 weeks	
Wu et al^28^	TACE	EPI (10-50 mg), gelatin sponge or polyvinyl alcohol particles.	N/A	Until the occurrence of untreatable progression or intolerant treatment-related toxicity.
	TACE + HAIC	After TACE. OXA (100 mg/m^2^, 2 hours); leucovorin (200 mg/m^2^, 2 hours); 5-FU (400 mg/m^2^, 15 minutes); 5-FU (600 mg/m^2^, 22 hours); leucovorin (200 mg/m^2^, 2 hours), 5-FU (400 mg/m^2^, 15 minutes), 5-FU (600 mg/m^2^, 22 hours).	3-5 weeks	
Li et al^27^	TACE	EPI (30 mg/m^2^), carboplatin (200 mg/m^2^), and mitomycin C (4 mg/m^2^) mixed with 2-5 mL lipiodol, additional pure lipiodol (up to 20 mL).	4 weeks	N/A
	TACE + HAIC	TACE using EPI (30 mg/m^2^) with lipiodol (2-5 mL), followed by pure lipiodol. HAIC: OXA (85 mg/m^2^) infusion for 2 hours; leucovorin (400 mg/m^2^) infusion for 2 hours; 5-FU (400 mg/m^2^) bolus and 5-FU (2400 mg/m^2^) continuous infusion for 46 hours or 5-FU (1200 mg/m^2^) continuous infusion for 23 hours.	4 weeks	
Huang et al^29^	TACE	Pirarubicin (60 mg), drug-eluting bead.	4 weeks	For the patients with emergence of new intrahepatic lesion, D-TACE–HAIC, or DEB–TACE would be performed for the residual viable primary tumor and the new intrahepatic lesion, but the treatment would be discontinued if it failed to achieve objective response.
	TACE + HAIC	After TACE. OXA (85 mg/m^2^, 2 hours); leucovorin (400 mg/m^2^, 2 hours); 5-FU (400 mg/m^2^, bolus infusion, and then 2400 mg/m^2^, 46 hours).	4 weeks	

CF, folinic acid; CT, computed tomography; DEB, drug-eluting beads; EPI, epirubicin; 5-FU, 5-­fluorouracil; HAIC, hepatic arterial infusion chemotherapy; n/a, not available; OXA, oxaliplatin; PVA, polyvinyl alcohol particles; TACE, transcatheter arterial chemoembolization.

**Table 3.** Subgroup Analysis of All Included Studies on Progression-Free Survival, Overall Survival, Complete Response, Partial Response, Stable Disease, and Progressive Disease

Outcomes	Subgroup	Studies	HR/RR and 95% CI	*P*	Heterogeneity
PFS	HCC with PVTT	2	0.67 [0.46, 0.98]	.04	*I*^2^ = 0
	Various unresectable HCC	4	0.36 [0.26, 0.50]	< .00001	*I*^2^ = 42%
OS	HCC with PVTT	2	0.57 [0.40, 0.80]	.001	*I*^2^ = 0
	Various unresectable HCC	3	0.60 [0.44, 0.82]	.002	*I*^2^ = 13%
CR	HCC with PVTT	2	4.20 [0.74, 23.91]	.11	*I*^2^ = 0
	Various unresectable HCC	5	1.79 [1.08, 2.98]	.02	*I*^2^ = 0
PR	HCC with PVTT	2	3.77 [0.75, 19.04]	.11	*I*^2^ = 55%
	Various unresectable HCC	5	1.89 [1.10, 3.25]	.02	*I*^2^ = 70%
SD	HCC with PVTT	2	0.92 [0.66, 1.28]	.63	*I*^2^ = 0
	Various unresectable HCC	5	0.80 [0.54, 1.19]	.27	*I*^2^ = 46%
PD	HCC with PVTT	2	0.58 [0.26, 1.26]	.17	*I*^2^ = 57%
	Various unresectable HCC	5	0.41 [0.29, 0.59]	<.00001	*I*^2^ = 0
ORR	HCC with PVTT	2	4.31 [0.92, 20.22]	.06	*I*^2^ = 64%
	Various unresectable HCC	5	2.07 [1.31, 3.26]	.002	*I*^2^ = 74%
DCR	HCC with PVTT	2	1.38 [1.05, 1.81]	.02	*I*^2^ = 14%
	Various unresectable HCC	5	1.32 [1.15, 1.51]	<.0001	*I*^2^ = 42%

CI, confidence interval; CR, complete response; HCC, hepatocellular carcinoma; HR, hazard ratio; OS, overall survival; PD, progressive disease; PR, partial response; PVTT, portal vein tumor thrombosis; SD, stable disease.

**Supplementary Table 1.** The Quality Assessment of the Included Studies in the Meta-Analysis

Study	Selection				Comparability	Outcome			Total
Resource	Representativeness of the exposed cohorts	Selection of the non-exposed cohorts	Ascertainment of exposure	Demonstration that outcome of interest		Ascertainment of outcome	Length of follow-up	Adequacy of follow-up	
Kim et al^23^	★	★	★	-	★	-	★	-	★★★★★
Gao et al^24^	★	★	★	★	★★	-	-	★	★★★★★★★
Guo et al^25^	★	★	★	-	★★	★	★	-	★★★★★★★
Liu et al^27^	★	★	★	★	★★	-	★	★	★★★★★★★★
Wu et al^28^	★	★	★	-	★	-	-	★	★★★★★
Li et al^26^	★	★	★	★	★★	★	★	-	★★★★★★★★
Huang et al^29^	★	★	★	★	★	★	★	★	★★★★★★★★

**Supplementary Table 2.** The Inclusion and Exclusion Criteria of Studies Included in the Meta-Analysis

Study	Inclusion criteria	Exclusion criteria
Kim et al^23^	Patients with HCC and hepatic vein invasion and Child-Pugh class A.	(a) ChildPugh class B or C; (b) previous treatment including surgical resection, radiofrequency ablation, and ethanol injection; (c) presence of ascites; and (d) presence of an additional malignancy.
Guo et al^25^	Eligible patients were aged 20 years or older with unresectable intermediate or advanced HCC, diagnosed according to the AASLD criteria of conclusive contrast-enhanced ultrasonography and magnetic resonance imaging without biopsy. They were administered TAE + HAIC or TACE and had an Eastern Cooperative Oncology Group performance (ECOG) status of 0–2 and tolerant liver function (Child–Pugh class ≤ B).	Unmeasurable lesions at baseline according to modified Response Evaluation Criteria in Solid Tumors (RECIST version 1.1) guidelines, for example, small lesions (≤10 mm in any dimension); diffusive lesions or tumors with obscure boundary; and incomplete patient data.
Liu et al^27^	(1) aged 18–85 years and had an adequate bone marrow count, which was defined as a white blood cell count > 3.0 × 109 /L or an absolute neutrophil count >1.5 × 109 /L, a platelet count of 60 × 109 /L, hepatic alanine aminotransferase (ALT) and aspartate aminotransferase (AST) levels ≤ 5 times the upper limit of normal, a serum creatinine level ≤ 2.0 mg/dL and a renal creatinine clearance ≤ 1.5 times the upper limit of the normal, an international normalized ratio ≤ 1.5, a Child–Pugh grade A or B, at least one measurable intrahepatic lesion according to the modified response evaluation criteria in solid tumors (mRECIST), and an Eastern Cooperative Oncology Group − Performance Status (ECOG − PS) ≤ 2; adequate collateral circulation from the anterior circulation must be indicated, when the portal tumor thrombus completely filled the major portal vein.	(1) patients with prior or concomitant malignancies, (2) those with diffuse lesions of HCC and with upper gastrointestinal bleeding history or ascites, (3) a left ventricular ejection fraction ≤ 45%, (4) patients with missing data on their first imaging assessments, and (5) those who were lost to follow-up.
Wu et al^28^	(1) patients aged 2070 years; (2) patients diagnosed as HCC based on the American Association for the Study of Liver Diseases guidelines; (3) patients diagnosed as PVTT complications according to the enhancement of contrast in the arterial phase and washout in the portal venous phase images of CT; (4) patients with tolerable liver function (ChildPugh score 56) at admission; (5) treatment naïve;(6) patients who refused to receive sorafenib treatment due to financial problems or other causes.	(1) patients whose baseline data was not complete; (2) patients with Eastern Cooperative Oncology Groupperformance status (ECOGPS) >2; (3) patients with unmeasurable lesions, such as diffusive ones, according to the modified Response Evaluation Criteria in Solid Tumors (RECIST version 1.1) criteria; (4) patients with Vp0 Vp3 in Japanese Vp classification, type I0II or IV in Cheng’s classification, hepatic vein tumor thrombosis, inferior vena cava tumor thrombosis, or atrium tumor thrombosis; (5) patients who received substandard treatments plus sorafenib, ablation, etc.; (6) patients with significant complications in the cardiopulmonary or nervous systems.
Gao et al^24^	(1) male or female patients 1880 years of age; (2) inoperable HCC patients (i.e., the surgical department eliminated a surgical option) without extrahepatic metastasis, including patients who had undergone surgery but suffered recurrence; (3) ChildPugh grade A or B for liver function; (4) Barcelona staging (BCLC staging) of hepatic lymph node metastasis (N1) and distant metastasis (M1), except for patients in stage A, B or C; (5) an Eastern Cooperative Oncology Group (ECOG) patient state (PS) grade of 0 or 1; (6) patients who had enough reserve functions in the liver, kidney and medulla ossium; and (7) and an estimated survival time ≥ 12 wk.	(1) the development or simultaneous development of other histological tumors; (2) patients who had undergone liver transplant surgery or received any other prior antitumor treatments, including interferon (IFNα), systemic chemotherapy, and sorafenib; (3) patients who had developed severe coronary heart disease, severe arrhythmia requiring treatment with medicines other than a β receptor blocking agent or digoxin, severe active infection (> grade 2, NCI CTCAE v3.0 criteria), combined HIV infection, renal insufficiency [creatinine (Cr) level > 2 mg/dL], unconsciousness (including patients with a history of epilepsy), severe allergic constitution, or allergy to contrast media; (4) women who were pregnant or lactating at the time of enrollment; (5) ECOG grading, PS > 2; (6) ChildPugh grade C for liver function; (7) BCLC staging, stage 0 or D; (8) the tumor volume accounted for > 70% of the liver volume; and (9) portal vein thrombosis with no obvious collateral circulation established.
Li et al^26^	(a) classified as the Barcelona clinic liver cancer (BCLC) stage A or B; (b) the tumor was not amenable to radical surgical resection, due to insufficient surgical margin, after assessment by 2 experienced hepatobiliary surgeons; or would have an estimated <30% residual liver volume (FLV) after resection; (c) classified as Child–Pugh Grade A.	(a) a previous history of HCC treatment; (b) signs of vascular invasion or distant metastasis on imaging; (c) severe underlying cardiac, pulmonary, or renal diseases; or (d) a second primary malignancy.
Huang et al^29^	1) the maximum lesion accessed larger than 5 cm on dynamic CT or MR images obtained within 7 days before treatment, 2) age between 18 and 75 years, 3) Eastern Cooperative Oncology Group performance status of 0 or 1, 4) Child–Pugh class A liver disease, and 5) adequate organ function, with hemoglobin ≥8.5 g/dL, white blood cell count ≥3.0 × 109 /L, neutrophil count ≥1.5 × 109 /L, platelet count ≥75 × 109 /L, aspartate transaminase and alanine transaminase ≤5 × upper limit of the normal, and creatinine clearance rate of ≤1.5 × upper limit of the normal.	1) had tumor invasion in bilateral first-order portal vein branch, the main trunk of portal vein, or inferior vena cava; 2) had evidence of extrahepatic metastasis before treatment; 3) had previously undergone sorafenib or lenvatinib therapy, systemic chemotherapy, HAIC, or TACE; 4) currently had or had a history of malignant tumors in addition to HCC; 5) had severe medical comorbidities including severe cardiopulmonary dysfunction and coagulation disorders (international normalized ratio ≥1.5); or 6) had a follow-up less than 3 months.

AASLD, the American Association for the Study of Liver Disease; HAIC, hepatic arterial infusion chemotherapy; HCC, hepatocellular carcinoma; PVTT, portal vein tumor thrombosis; RECIST, response evaluation criteria in solid tumors; TACE, transcatheter arterial chemoembolization; TAE, transcatheter arterial embolization.
